# The mechanism by which MALAT1/CREG1 regulates premature rupture of fetal membrane through autophagy mediated differentiation of amniotic fibroblasts

**DOI:** 10.1016/j.ncrna.2025.04.004

**Published:** 2025-04-10

**Authors:** Xiaomei Huang, Ting Huang, Aixing Chen, Yong Shao

**Affiliations:** a Department of Obstetrics and Gynecology, The First Affiliated Hospital of Chongqing Medical University, Chongqing, 400016, China; b Department of Obstetrics and Gynecology, Chongqing Health Center for Women and Children, Women and Children's Hospital of Chongqing Medical University, Chongqing, 400016, China

**Keywords:** Premature rupture of fetal membrane, Metastasis-associated lung adenocarcinoma transcript 1, Autophagy, Fibroblasts

## Abstract

**Background:**

Premature rupture of fetal membrane (PROM) is one of the main causes of premature delivery. The amniotic membrane plays a major role in bearing weight, and amniotic fibroblasts play an important role. The purpose of this study was to explore the scientific problems associated with amniotic membrane repair by intervening with fibroblasts to provide evidence for the clinical treatment of PROM.

**Methods:**

This research group conducted experiments on fetal membrane tissue via single-cell sequencing, Sirius staining, fluorescence staining and Raman spectroscopy to explore changes in fetal membrane structure and verified key targets and pathways in clinical tissues and primary fibroblasts through WB, PCR, RNA Pulldown, RIP and molecular docking experiments.

**Results:**

The fetal membrane structure in the PROM group was obviously damaged, and the amniotic fibroblasts were activated and autophagy was activated, and the activated autophagy promoted the activation of fibroblasts. The expression of Metastasis-Associated Lung Adenocarcinoma Transcript 1 (MALAT1) was significantly increased in amniotic fibroblasts. RNA PULL DOWN and molecular docking results suggested that MALAT1 binds to human E1A promoter repressor 1 (CREG1) and promotes autophagy.

**Conclusions:**

By interacting with CREG1, MALAT1 can increase the expression of CREG1, regulate the expression of autophagy-related molecules, mediate the differentiation of amniotic fibroblasts into myofibroblasts, participate in amniotic repair, and promote the repair of PROM fetal membrane tissue.

## 1 Introduction

Premature rupture of membranes (PROM) occurs naturally before delivery and is one of the main causes of premature rupture [1]. Premature birth is one of the leading causes of infant mortality, and is a syndrome induced by a variety of pathological processes that can lead to intraventricular hemorrhage in infants, neonatal respiratory distress syndrome, and underdeveloped fetal lungs, among other complications [2,3]. It is one of the primary causes of obstetric and neonatal complications. Current clinical treatments for PROM primarily focus on controlling infection with antibiotics, maintaining pregnancy, and promoting lung maturation through symptomatic treatment [3]. There is no definitive protocol for controlling PROM to date.

Research has revealed that fetal membranes are composed of an amniotic layer and a chorionic layer [4], which are connected through the extracellular matrix (ECM). The amniotic layer is further divided into the epithelial cell layer, basement membrane layer, compact layer, fibroblast layer, and spongy layer. The chorionic layer is stratified into the cellular layer, reticular layer, pseudobasal layer, and trophoblast layer. The amniotic layer governs the mechanical behavior of the fetal membranes and serves as a structural barrier [5], whereas the chorionic layer acts as an immunological buffering barrier, preventing the degradation of the amniotic layer and protecting the fetus from the maternal immune system [6]. The amniotic layer is more robust, rigid, and extensible than the chorionic layer, and plays a primary load-bearing role within the fetal membranes [[6], [7], [8]]. Fibroblasts, located between the compact and spongy layers of the amniotic layer, produce components such as collagen, proteoglycans, and ECM [9], constituting a significant part of the amniotic layer. It has been reported that dermal fibroblasts can invade the healing site, promoting repair and regeneration [10]. Once located at the site of injury, fibroblasts are activated and differentiate into myofibroblasts, which produce extracellular ECM proteins to promote wound closure, such as collagen and fibronectin (FN) [11]. Proteins synthesized by fibroblasts are key to wound healing. For example, after an acute myocardial infarction, type III collagen produced by fibroblasts helps repair the damage [12].

Our experimental investigations revealed that, compared with those in normal fetal membrane tissues, the amniotic epithelial cells in PROM tissues are not closely connected, and there are gaps in the structure of the amniotic layer [13]. RICHARDSON et al. [14] reported that there are certain "morphological change areas" in fetal membranes with PROM (where fibroblasts and the spongy layer near the rupture site have fewer collagen fibers and less tissue) and microcracks (from the amnion through the ECM to the chorionic layer). These may be formed by cell shedding or wrinkling, and the degradation of the basal membrane and matrix collagen results in the formation of channels. Cracks can serve as pathways for the leakage of amniotic fluid and the migration of inflammatory cells and microorganisms, which may be early events of PROM. This finding indicates that the formation of microcracks in the amnion is closely related to the occurrence and development of PROM. Therefore, finding strategies for repair of microcracks may effectively intervene in the early rupture of fetal membranes.

Our research previously performed single-cell sequencing analysis on fetal membrane tissues extracted from patients with PROM and conducted multitiered validation involving clinical specimens and primary fibroblasts. The amniotic fibroblasts in PROM were activated,and autophagy was activated. Activated autophagy can promote the activation of fibroblasts, and once amniotic fibroblasts are activated into myofibroblasts, they can facilitate tissue repair. Data analysis revealed a significant increase in the expression of Long Non-Coding RNA Metastasis-Associated Lung Adenocarcinoma Transcript 1 (MALAT1) in amniotic fibroblasts. The results from RNA pull-down and molecular docking suggest that MALAT1 binds to human E1A activation gene inhibitor 1 (CREG1), promoting autophagy. In this study, we focused on amniotic fibroblast MALAT1 as our subject of investigation, using the autophagy mediated by MALAT1 and its associated protein CREG1 as a point of entry. We delved into the regulatory mechanisms underlying the differentiation of fibroblasts and myofibroblasts and further analyzed their role in the occurrence and progression of PROM. Our findings suggest that MALAT1, through its interaction with CREG1, enhances the expression of CREG1, thereby regulating autophagy-related molecules that mediate the differentiation of amniotic fibroblasts into myofibroblasts, participating in the repair of the amnion and subsequently promoting the repair of PROM fetal membrane tissue. This research may provide new insights into the mechanisms of amniotic membrane repair and offer novel targets for the prevention and treatment of PROM.

## 2 Materials and methods

### 2.1 Collection of human fetal membrane samples

Clinical specimens were obtained from the First Affiliated Hospital of Chongqing Medical University in strict accordance with relevant ethical guidelines for clinical research. Written informed consent was obtained from all participants, and the study protocol was approved by the Ethics Committee of the First Affiliated Hospital of Chongqing Medical University (No. 2022-160). The blood present on the surface of the fetal membranes was thoroughly washed with phosphate-buffered saline (PBS). Following this, the entire fetal membrane was excised, and the amniotic membrane and chorionic membrane were manually separated. The fetal membrane and amniotic membrane were fixed in 4 % paraformaldehyde and 2.5 % glutaraldehyde, while the remaining tissues were preserved at −80 °C.

Fetal membranes of pregnant women who underwent cesarean section at the First Affiliated Hospital of Chongqing Medical University from January 2023 to January 2024 were collected. The case group (PROM, n = 20) comprised individuals who experienced PROM at ≥30 weeks of gestation, and were diagnosed on the basis of a positive fetal fibronectin test in the cervical vaginal fluid. The NORMAL group consisted of normal pregnancies (NORMAL, n = 20) without any complications. All participants had no preeclampsia, hypertension, multiple pregnancies, asthma, cardiovascular disease, diabetes, fetal growth restriction, fetal abnormalities, sexually transmitted disease, thyroid disease, or uterine malformation. Details regarding the pregnant women included for single-cell sequencing are presented in Table 1.

**Table 1:** Patient information from single-cell sequencing.

Sample number	Age of delivery (years)	Newborn weight (g)	Apgar score	Diagnosis
N1	25	3010	10-10-10	1. Scarred uterus (previous cesarean section); 2. G2P1 of pregnancy to be delivered
N2	28	2960	10-10-10	1. Scarred uterus (previous cesarean section); 2. G4P1 of pregnancy to be delivered
N3	30	3115	10-10-10	1. Breech position; 2. G1P0 of pregnancy to be delivered
P1	26	2540	9-10-10	1. Premature rupture of fetal membrane before term; 2. Pregnancy with uterine scar; 3. G4P1 of threatened preterm delivery
P2	29	2860	9-10-10	1. Premature rupture of membranes; 2. G1P0 of threatened preterm delivery
P3	32	2930	10-10-10	1. Premature rupture of membranes; 2. G1P0 of threatened preterm delivery

### 2.2 Single-cell sequencing

The fresh tissue is kept in the tissue preservation solution, and the tissue is rinsed, digested, dissociated and cracked red. Trypan blue (Sigma) staining was employed to assess cell viability and quantify cell counts were quantified via microscopy. For single-cell RNA sequencing (scRNA-seq), a single-cell suspension was prepared, and the cell concentration was adjusted to 1 × 10^5^ cells/mL. The single-cell suspension was then loaded onto the GEXSCOPE™ microfluidic chip, and the single-cell RNA library kit was used to construct the scRNA-seq library following the manufacturer's instructions. The raw sequencing data were processed to generate a gene expression matrix. ClusterProfiler software was used to perform Gene Ontology (GO) enrichment analysis, identifying significantly expressed genes associated with specific biological functions or pathways.

### 2.3 Hematoxylin-eosin (HE) staining

Fetal membrane tissues were collected and prepared into paraffin sections according to standard protocols. The sections were dewaxed in xylene, rehydrated in graded alcohol, and stained with hematoxylin. After rinsing, differentiation was achieved using 0.1 % acid ethanol, followed by eosin staining. The sections were subsequently washed in alcohol, dehydrated, and cleared in xylene before being mounted with resin. Observations were made under an optical microscope, and images were captured.

### 2.4 Sirius staining

To quantitatively assess the collagen content within fetal membranes, paraffin sections were prepared with fetal membranes from the NORMAL and PROM groups. The sections were stained with Sirius Scarlet solution (Servicebio, G1018) for 1 h, followed by rinsing under running water. After dehydration, the samples were made transparent with a resin sealing glass slide, and a polarized light microscope and bright field microscope were used. Under bright field microscopy, collagen appears red, whereas polarized light microscopy allows for the differentiation of collagen types I (bright yellow) and III (green) on the basis of their respective colors, as well as an assessment of the molecular arrangement of the proteins [15].

### 2.5 Transmission electron microscopy (TEM)

To elucidate the ultrastructural alterations within the amniotic membrane and cells, a comprehensive analysis was conducted using TEM. The samples were infused with 2.5 % glutaraldehyde, fixed with 1 % osmium tetroxide (0.1 M, pH 7.2), dehydrated in a graded series of ethanol (70, 80, 90, and 100 %), clarified in toluene, and embedded in Epon 812 at 60 °C for 24 h. A semithin section of 1 μm was cut, stained with toluidine blue, and observed in the correct direction under an optical microscope. For TEM analysis, ultrathin sections (60 nm) were prepared and contrasted with uranyl acetate and lead citrate, facilitating high-resolution imaging of the cellular ultrastructure.

### 2.6 Immunofluorescence

Immunofluorescence was conducted on the amniotic membrane or cells fixed in 4 % paraformaldehyde for 30 min, followed by permeabilization in 0.5 % Triton X-100 for 15 min. The amniotic membrane or cells were transferred to serum containing 5 % BSA on PBS and sealed at room temperature for 2 h. These samples were then incubated with primary antibody at 4 °C overnight. The next day, the sample was washed twice in PBS containing 1 % BSA and then incubated with the corresponding secondary antibody for 2 h. The antibodies used were cytokeratin 19 (GB12198, Servicebio, China), Vimentin (ab137321, Abcam, UN), and α-SMA (1:200, GB111364, Servicebio, China). The samples were then stained with Hoechst/DAPI for 10 min and photographed using confocal or fluorescence microscopy.

### 2.7 Raman spectroscopy

Raman spectroscopy, a label-free, rapid, and noninvasive optical detection technique, has garnered significant interest in the realm of biomedical research. It is increasingly recognized as a potentially valuable clinical tool in cancer diagnostics, enabling the identification of subtle biochemical alterations in biological samples such as tissues, cells, serum, urine, and saliva that are indicative of cancer progression [16]. In the context of hypertensive pregnancies, Raman spectroscopy has demonstrated a certain degree of predictive efficacy in the analysis of serum samples. However, its application in the detection of PROM has not yet been explored [17]. A Renishaw in Via micro-Raman spectroscopy system was used to record the Raman spectra of the amniotic wax samples. Each spectrum was recorded with a collection time of 10 s, and the data were accumulated three times to increase the signal-to-noise ratio. LabSpace 6.0(HORIBA Scientific, Japan) was used for smoothing, baseline correction and normalization of the spectral data to obtain more realistic Raman signals on slices. Subsequent statistical analyses, encompassing the calculation of mean Raman spectra and principal component analysis (PCA), were performed using Origin 2021 software.

### 2.8 Extraction and culture of human amniotic fibroblasts

After the chorionic membrane was peeled, the amniotic tissue was digested twice with 0.125 % trypsin and then vigorously washed with PBS to remove the remaining epithelial cells. The remaining amniotic tissue was digested with 0.1 % collagenase (Sigma) to release fibroblasts from the mesenchymal tissue. After centrifugation of the digestion medium, the precipitates were resuspended and stratified on a percoll gradient to further purify fibroblasts stratified by approximately 40 % percoll. The isolated amniotic fibroblasts were cultured in DMEM supplemented with 10 % fetal bovine serum and 1 % penicillin and streptomycin. The primary amniotic fibroblasts were spindle shaped, star shaped and polygonal and were distributed radially or whirly, and fibroblast-like clones could be observed. To confirm the cellular identity, immunofluorescence staining was employed, which targeted the mesenchymal cell marker vimentin and epithelial cell marker CK19. Autophagy induction: Cells were inoculated in a Petri dish, and once the cells reached confluence, they were cultured in serum-free medium (SS) for 4 days. Studies have shown that prolonged serum starvation can trigger autophagy [18]. Conversely, to inhibit autophagy, the cells were cultured in a Petri dish until confluence was reached, at which point chloroquine (40 μmol/L) was added to the culture medium for a duration of 48 h. Chloroquine, known for its ability to disrupt autophagic flux by inhibiting lysosomal function, was utilized to study the effects of autophagy inhibition on the cellular phenotype and function.

### 2.9 Cell transfection

SiRNAs were purchased from Shanghai Shenggong Company and Siding Biology, 20 nM siRNA and Lipo3000 (L3000015, Thermo Fisher, USA) were used to transfect amniotic fibroblasts for 24 h. The following siRNAs were used in this study:

siMALAT1-1- sense: 5′-GAGGUGUAAAGGGAUUUAUTT-3′

siMALAT1-1-antisense: 5′-AUAAAUCCCUUUACACCUCTT-3′

siMALAT1-2- sense: 5′-CACAGGGAAAGCGAGTGGTTGGTAA-3′

siMALAT1-2-antisense: 5′-TTACCAACCACTCGCTTTCCCTGTG-3′

CREG1-sense: 5′-CAGAAAUGGAUAUUGCAAATT-3′

CREG1-antisense: 5′-UUUGCAAUAUCCAUUUCUGTT-3′

mCherry-GFP-LC3 adenovirus (C3011) was purchased from Beyotime Company. Amniotic fibroblasts at a 50 % confluence were infected with adenovirus at an MOI of 20 for 24 h and then incubated in medium for 48 h. Autophagy flow was observed with Olympus confocal microscope (Japan).

### 2.10 RNA pulldown

The RNA Pulldown is performed according to the manufacturer's instructions. The experimental procedures included RNA secondary structure formation, probe bead preparation, total cell protein extraction, nucleic acid removal, RNA binding protein collection and mass spectrometry.

Primer sequences: foward: TGTGTGCCAATGTTTCGTTT,

Reverse: AGGAGAAAGTGCCATGGTTG;

MALAT1 probe sequence: TCTCAATCCTGAAATCCCCTAGGGAAG,

NC probe sequence: TTGTGCCCATTAACATCACCATCTAA.

### 2.11 RNA immunoprecipitation (RIP)

The magnetic beads were coated with 5 μg of antibodies, and then the cell lysate was added to the magnetic beads coupled with CREG1 antibodies and IgG antibodies. The magnetic bead-protein-RNA complex was washed with RIP washing buffer. RNA was extracted by adsorption column extraction. The cDNA was then generated using PrimeScript RT kit and MALAT1 expression was detected by qPCR.

### 2.12 Fluorescence in situ hybridization (FISH)

A FISH kit (C10910, RiboBio, China) was used to fix the cells, permeabilize them, denature them, bake them, rinse them, rinse them naturally dry them, rest them with DAPI, and observe the results via confocal microscopy.

### 2.13 Western blot(WB)

The amniotic tissue was cut into small pieces, homogenized on ice with a tissue grinder, and then cleaved in a RIPA buffer containing a protease phosphatase inhibitor (P1261, Solarbio), and the cells were cleaved directly in a RIPA buffer containing both a phosphatase inhibitor and a protease inhibitor. After quantification via the BCA method, each sample (including 20 g protein) was separated via polyacrylamide gel electrophoresis and transferred to a PVDF membrane. The blots were blocked with a rapid blocking solution (WB4600, NCM Biotech) for 10 min and then incubated overnight with a specific primary antibody at 4 °C. The antibodies were LC3B (1:1000, 12741, CST, USA), P62 (1:1000, AF5384, Affinity, USA), Beclin-1 (1:1000, AF5128, Affinity, USA), ATG5 (1:1000, DF6010, Affinity, USA), CREG1 (1:1000, A16081, ABclonal, USA), PEIF2AK3 (1:500, Catalog#: 20536-1-AP, Proteintech, USA), EIF2AK3 (1:500, Catalog#: HA210525, HUABIO, China), beta ACTIN (1:500, Catalog#: 20536-1-AP, Proteintech, USA) and α-SMA (1:1000, BF9212, Affinity, USA). The membrane was then washed and incubated with an HRP-conjugated for rabbit/mouse secondary antibody incubated at room temperature for 1 h. The band signals were visualized and analyzed using enhanced chemiluminescence reagents (Millipore Sigma) and Vilber Fusion imaging system (Fusion FX5 Spectra, France).

### 2.14 Quantitative real-time polymerase chain reaction (q-PCR)

According to the manufacturer's protocol, total RNA was extracted from the tissue using TRIzol reagents, cDNA was synthesized using the Evo M-MLV RT Mix Kit, and qPCR was performed using the SYBR Premix Ex Taq and LightCycler™ 96 instruments. The primer information is as follows:

H-GAPDH-F: 5′-GCACCGTCAAGGCTGAGAAC-3′;

H-GAPDH-R: 5′-TGGTGAAGACGCCAGTGGA-3′;

H-MALAT1-F: 5′-GCTCTGTGGTGTGGGATTGA-3′;

H-MALAT1-R: 5′-GAGAAGTGGCAAAATGGCGG-3′;

H-CREG1-F: 5′-TCCCAAACCACTCTCCAC-3′;

H-CREG1-R: 5′-GCCATGTTCTCTTCCCTCT-3′.

### 2.15 Molecular docking

The CREG1 protein, comprising 220 amino acids, has a molecular weight of approximately 24.06 kDa. Interestingly, the nucleic acid sequence of MALAT1 spans more than 8000 nucleotides, with only the sequence from 1 to 76 characterized, which adopts a triple helix structure. Notably, the CREG1_HUMAN protein is known to exist as a dimer, with two dimers associated with form a functional tetramer. In this study, we propose to computationally dock the CREG1_HUMAN dimer structure with the MALAT1 triple helix structure, utilizing software to predict the binding interface of this interaction. This approach provides insights into the molecular mechanisms underlying their interaction, potentially revealing novel regulatory mechanisms in cellular processes.

### 2.16 Data independent acquisition (DIA)

Appropriate samples were taken and quantified by BCA method. Protein samples (15 μg) were subjected to SDS-PAGE and subjected to enzymolysis. After drying, the peptide was redissolved in 0.1 % FA and the concentration of the peptide was determined for LC-MS analysis. DIA mass spectrometry data collection: Appropriate peptide segments were taken from each sample and chromatographic separation was performed using a chromatographic system. The peptides were separated and analyzed by DIA mass spectrometry. By combining all mass spectrum data with software, DIA mass spectrum data database retrieval and protein DIA quantitative analysis were completed.

### 2.17 Determination of cell migration

To assess cellular migration capabilities during wound healing assays, cells were seeded into a 6-well plate and maintained in a humidified incubator at 37 °C with 5 % CO_2_ to achieve confluence, typically reaching approximately 100 % cell confluence. The wound was made with a sterile pipette suction, and the cell debris was removed by washing with PBS. At predefined time points, specifically 0, 12, and 24 h post wounding, the cells were examined using an optical microscope to capture images of the wounded area. These images were subsequently subjected to quantitative analysis to evaluate the rate and pattern of cell migration into the denuded zone.

### 2.18 Transwell migration assay

Transwell migration assay was performed using a 24-well chamber with 8 μm pore polycarbonate membranes (BIOFIL, China). The bottom chambers were filled with 600 μL DMEM supplemented with 10 % fetal bovine serum. The 200 μL of cell suspension in DMEM containing 1 × 10^5^ cells was added to the upper chamber. Cells were further incubated in an incubator with 5 % CO_2_ at 37 °C for 24 h. Cells were fixed with 4 % paraformaldehyde. After staining with 0.1 % crystal violet for 15 min, the cells were rinsed with PBS. Images were then captured with a microscope. ImageJ is used to count the number of migrating cells.

### 2.19 Cell counting kit-8 (CCK8) assay

Cell viability was assessed using the Cell Counting Kit-8 (CCK8, Beyotime Biotech, Shanghai, China). Cells were seeded in a 96-well plate at a density of 1 × 10^3^ cells per well. After 8, 12 and 24 h of exposure to the respective treatments, 10 μL of CCK8 solution was added to each well, followed by a 2-h incubation. Absorbance was measured using a microplate reader (Bio-Rad, USA).

### 2.20 Statistics

All the data were analyzed and visualized with GraphPad Prism 8.0. The number of samples (n) is shown in the legend. Comparisons between two groups were performed using an unpaired T-test. All the data are expressed as the means ± SEMs, and P < 0.05 was considered statistically significant.

## 3 Results

### 3.1 Abnormal tissue structure of the PROM

**Fig. 1:**
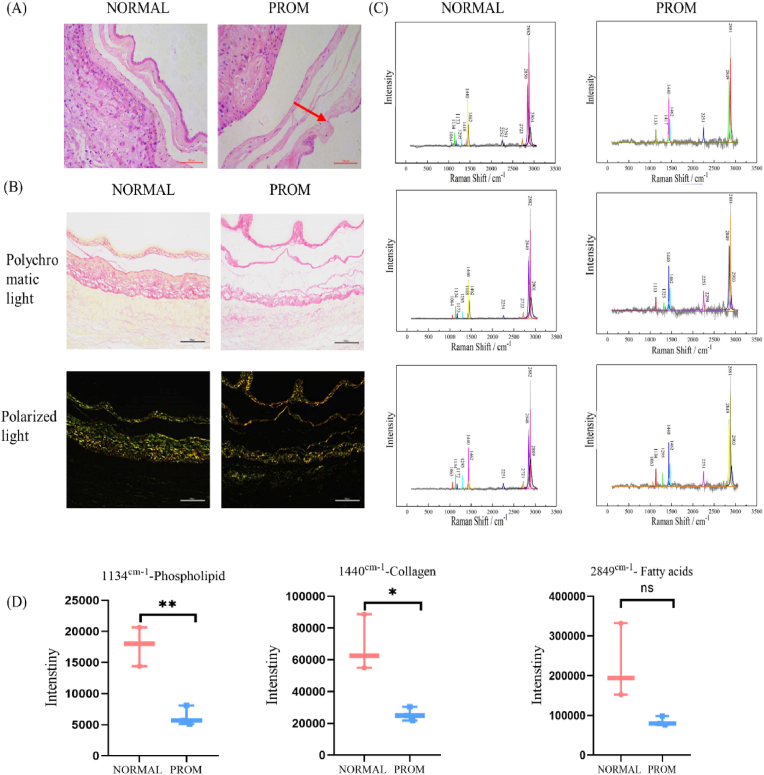
Structural anomaly of premature rupture of membranes. (A) Representative images of HE staining of fetal membrane tissue, and red arrows indicate defects, 20X. (B) Representative images of Sirius staining of fetal membrane tissue, 20X. (C) Raman spectrogram of amniotic tissue. (D) Statistical Raman spectra of amniotic tissue (phospholipids, collagen, and fatty acids).

The HE staining of the fetal membrane tissue revealed gaps in the amniotic epithelial cells in the PROM group, and the connections were not tight (Fig. 1A). The results of Sirius staining indicated that the collagen bundles were very compact and highly refractive in NORMAL tissues. In contrast, PROM tissues showed a marked reduction in birefringence intensity, suggesting a disintegration of the collagen architecture, with fragmented and irregular collagen frameworks, and dispersed collagen structures, indicative of a disarray of the collagen protein organization (Fig. 1B).

In this study, Raman spectroscopic analysis was conducted on amniotic membrane samples within the characteristic spectral region spanning 300–2850 cm^−1^. Distinct spectral peaks at 1064, 1134, 1173, 1295, 1418, 1440, 1462, 2252, 2291, 2723, 2850, 2882 and 2901 cm^−1^ were observed for the amniotic membrane tissues from both groups (Fig. 1C). The assignment of each spectral peak is detailed in Table 2. The difference spectra indicate that the Raman spectra of the PROM samples are significantly different from those of the NORMAL samples. The strength of the samples from patients with PROM ranging from 2850 cm^−1^, 1144 cm^−1^ and 1133 cm^−1^ was lower than that of the NORMAL samples (Fig. 1D), indicating decreased collagen, monounsaturated fatty acids, and phospholipids in the active components of the PROM samples. These spectral differences reflect the molecular compositional alterations in PROM amniotic membranes as the disease progresses.

**Table 2:** Representative products of spectral peaks.

Spectral peak	Representative product
1064	Lipid
1134	Phospholipid
1173	Nucleic acid
1295	Dioxy phosphate
1418	Cholesterol
1440	Collagen
1462	Disaccharide
2850	Monounsaturated fatty acids
2882	Lipids and proteins

### 3.2 Results of single-cell transcriptome sequencing of PROM

**Fig. 2:**
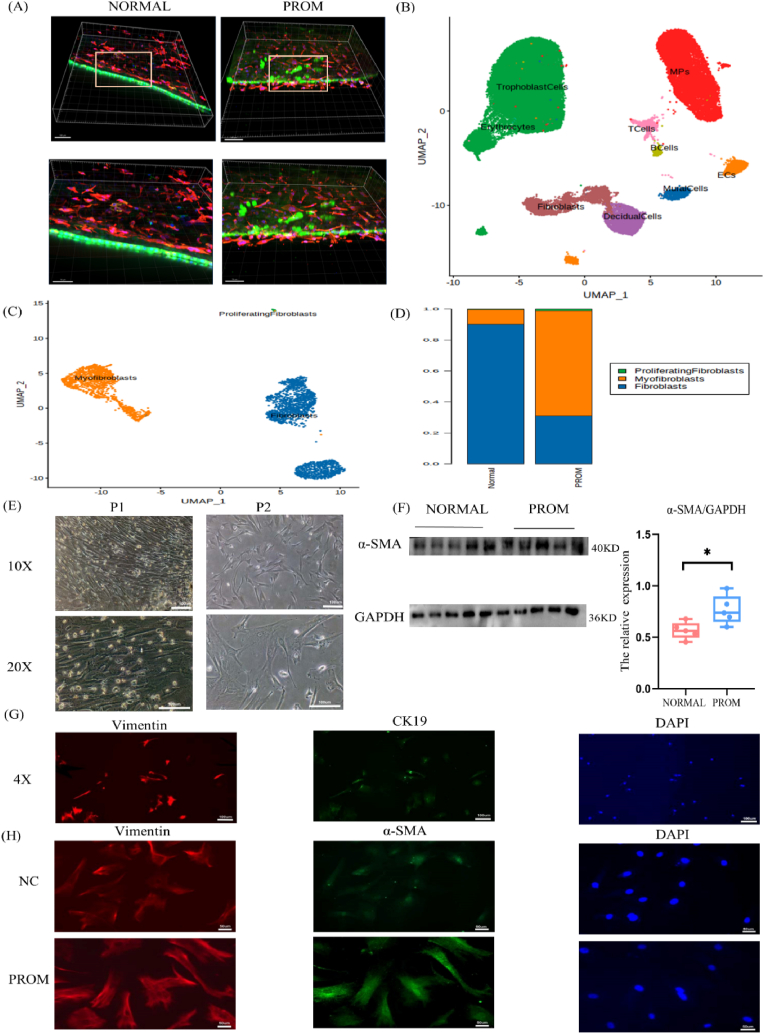
Analysis and verification of single-cell RNA sequencing data. (A) Representative images of fluorescent confocal 3D imaging of amniotic tissue showing structural abnormalities of amniotic cells in the PROM group. Green: CK19 (epithelial cell marker); red: vimentin (fibroblast marker); blue: DAPI. (B) Nine cell subtypes were obtained by cluster analysis of fetal membrane cells. (C) UMAP of fibroblast clusters from the NORMAL group and PROM group, 3 subpopulations with a total of 3023 cells were obtained by detection clustering. They are fibroblasts, myofibroblasts and proliferating fibroblasts. (D) The bar chart shows the proportion of fibroblast clusters in the NORMAL and PROM groups. (E) Representative images of the morphology of primary amniotic fibroblasts. (F) WB results showing the expression of the α-SMA in the NORMAL and PROM groups. (G) Representative images of cellular immunofluorescence, 4X. Red: vimentin; green: CK19; blue: DAPI. (H) Representative images of cellular immunofluorescence, 20X. Red: vimentin; green: α-SMA; blue: DAPI.

The confocal 3D imaging of CK19 and Vimentin immunofluorescence within the amniotic tissue revealed disruptions in the amniotic epithelial junctions and a sparse distribution of fibroblasts in the PROM group (Fig. 2A). These observations suggest that compromised integrity of fibroblasts and epithelial cells within the amniotic tissue is associated with PROM.

Single-cell sequencing analysis of fetal membrane tissue identified a total of nine distinct cellular clusters, which were categorized and color-coded using a UMAP diagram. A comprehensive analysis was conducted on 45,127 cells across nine subtypes, including trophoblast cells, fibroblasts, mononuclear phagocytes, erythrocytes, decidual cells, endothelial cells, T cells, B cells, and Mural cells (Fig. 2B). Notably, trophoblast cells and fibroblasts presented relatively high levels, with fibroblasts being a crucial component of the amniotic layer [9]. Given the pivotal role of fibroblasts in tissue repair, as evidenced by their protein synthesis capabilities, an in-depth investigation of these cells is warranted. Fibroblasts were subdivided into three groups (fibroblasts, myofibroblasts and proliferating fibroblasts) (Fig. 2C). A comparative analysis with the NORMAL group indicated a reduction in fibroblasts within the PROM group. The proliferation of myofibroblasts (Fig. 2D) implies the activation of fibroblasts in response to PROM, potentially reflecting a reparative mechanism within the amniotic membrane during the disease process.

### 3.3 MALAT1 can promote fibroblast transformation

**Fig. 3:**
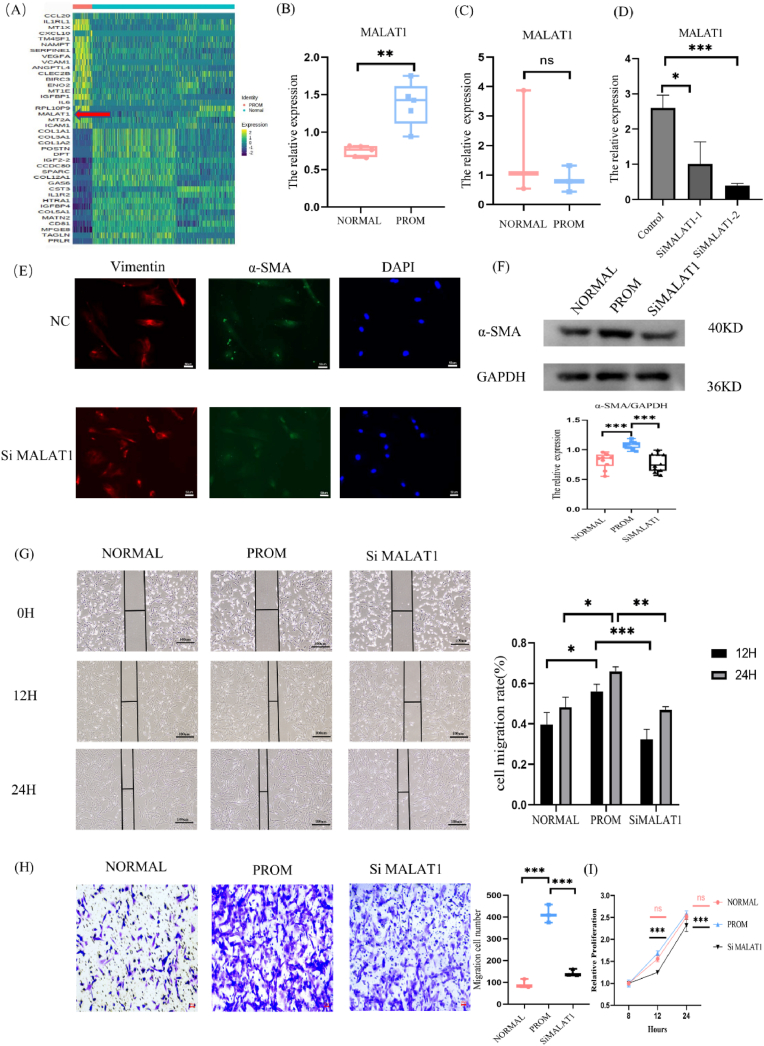
MALAT1 promotes fibroblast transformation. (A) Heat maps showed the top 20 up-regulated and down-regulated differentially expressed genes in NORMAL and PROM composed fibrocytes. (B) Q-PCR results indicating that the expression of MALAT1 in amniotic tissue was significantly increased, n = 5. (C) Q-PCR results indicating that there was no significant difference in the expression of MALAT1 in chorionic tissue, n = 3. (D) Q-PCR was used to detect the mRNA expression of cells after MALAT1 knockout, n = 3. (E) Representative images of cellular immunofluorescence, 20X. Red: vimentin; green: α-SMA; blue: DAPI. (F) Representative images and statistical plots of α-SMA expression detected by WB, n = 10. (G) Scratch maps and statistical results of 0, 12, and 24 h of exposure to amniotic fibroblasts, n = 7. (H) Representative images and statistical results of cell transwell migration experiment, n = 3. (I) Statistical results of cell CCK8 proliferation experiment, n = 3. The pink statistical line represents the statistics of PROM group and NORMAL group, and the black statistical line represents the statistics of PROM group and Si MALAT1 group.

Analysis of the upregulated genes in fibroblasts from single-cell sequencing data revealed a significant increase in MALAT1 expression within the PROM group (Fig. 3A). Quantitative PCR (qPCR) assessment of amniotic membrane samples further confirmed that MALAT1 expression was markedly elevated in the PROM group compared with the NORMAL group (Fig. 3B). However, no statistically significant difference in MALAT1 expression was detected in the chorionic membrane (Fig. 3C). Consequently, MALAT1 in amniotic fibroblasts was selected for further investigation.

To downregulate MALAT1 expression, small interfering RNA (siRNA) was used to knockdown MALAT1 expression. The mRNA expression levels of Control, SiMALAT1-1, and SiMALAT1-2 were measured to determine the knockdown efficiency of MALAT1 post-siRNA transfection in fibroblasts. The data demonstrated that, compared with the Control group,both siRNAs significantly reduced MALAT1 expression with SiMALAT1-2 exhibiting the lowest level of MALAT1 expression. Therefore, SiMALAT1-2 was chosen for subsequent MALAT1 knockdown experiments (Fig. 3D).

The expression of α-SMA in amniotic fibroblasts was assessed using WB. Compared with the NORMAL group, the PROM group presented significantly greater α-SMA levels, indicating myofibroblast activation. Notably, α-SMA levels decreased following MALAT1 knockout, suggesting that the activation of myofibroblasts in PROM can be inhibited by the loss of MALAT1 (Fig. 3F). Immunofluorescence analysis further corroborated these findings, revealing an increase in the fibroblast marker vimentin and a decrease in α-SMA levels after MALAT1 knockout in amniotic fibroblasts(Fig. 3E). These findings indicate that MALAT1 knockout can reverse fibroblast activation. Scratch experiments of fibroblasts revealed that the mobility of the PROM group at 12 and 24 h were higher than that of the NORMAL group, and the mobility decreased after MALAT1 was knocked down, demonstrating that MALAT1 can affect the migration function of cells (Fig. 3G). The transwell migration experiment also demonstrated that the number of cell migrations in the PROM group was higher than that in the NORMAL group, and the number of cell migrations decreased after MALAT1 knockdown (Fig. 3H). The CCK8 proliferation experiments showed no statistically significant difference between the PROM and NORMAL groups, but cell proliferation was significantly inhibited after MALAT1 knockdown (Fig. 3I), which proved that MALAT1 could affect the migration and proliferation of fibroblasts, but the migration movement may be more favorable in PROM.

### 3.4 Autophagy can promote fibroblast differentiation

**Fig. 4:**
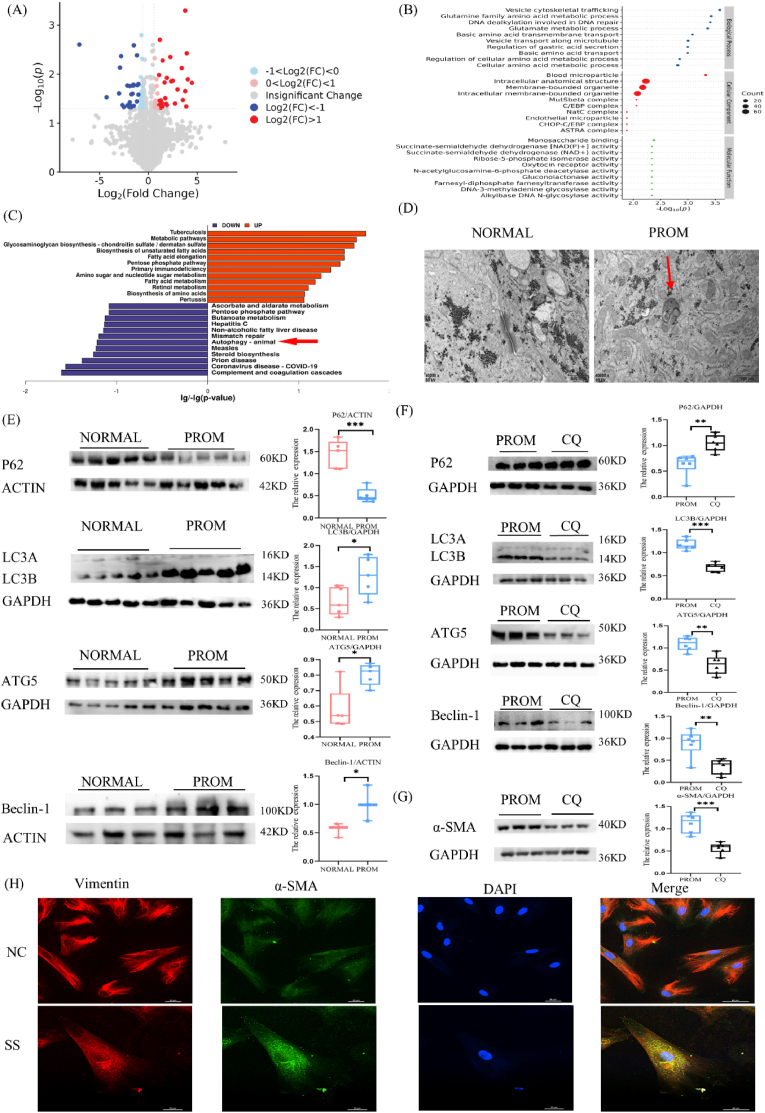
Relationship between autophagy and fibroblast transformation. (A) For the volcano map of the SiRNA vs NC group, the horizontal coordinate is the logarithmic transformation of FC, and the vertical coordinate is the negative logarithmic transformation of the p value. The red dots represent the significantly highly expressed proteins, and the darker the color is the higher the upregulation ratio. The blue dots represent significantly lower protein expression, and the darker the color is the greater the downregulation ratio. The gray dots represent non-differentially expressed proteins. (B) GO functional enrichment bubble diagram, showing the top 10 terms significantly enriched with differentially expressed under the three branches of BP, MF and CC. The horizontal coordinate is the negative logarithmic transformation of the enrichment significance p-value, and the vertical coordinate is the GO term. Each circle represents a term, and the size of the circle represents the count. (C) The KEGG pathway with the top 12 enrichment significance is represented by logarithmic (downregulated) and negative logarithmic (upregulated) transformation of p value at the horizontal coordinate, with blue columns representing downregulated protein enrichment pathways and red columns representing upregulated protein enrichment pathways (arrows indicating autophagy pathways). (D) Autophagosomes (arrow indicating autophagosomes) were observed in the TEM of amniotic tissue from patients with PROM. (E) WB results showing the expression of autophagy-related molecules P62, LC3B, ATG5 (n = 5) and Beclin-1 in amniotic tissue (n = 3). (F) WB results showing the expression of the autophagy-related molecules P62, LC3B, ATG5 and Beclin-1 in the PROM and CQ groups, n = 6. (G) WB results showing α-SMA expression in the PROM and CQ groups, n = 6. (H) Immunofluorescence staining of cells cultured in serum-free medium (SS), 40X. Red: vimentin; green: α-SMA; blue: DAPI.

After MALAT1 was knocked out, quantitative protein sequencing was conducted on the cells, revealing the upregulated and downregulated proteins (Fig. 4A). The top 10 differentially enriched proteins under the categories of Biological Process (BP), Molecular Function (MF), and Cellular Component (CC) are presented in Fig. 4B. Additionally, the KEGG pathway with the top 12 significantly enriched proteins is shown in Fig. 4C. Notably, the autophagy pathway appears to be downregulated, implying that the knockdown of MALAT1 hinders this pathway, thereby suggesting that MALAT1 plays a role in activating autophagy.

Through transmission electron microscope observation of amniotic tissue, autophagosomes were found in the amniotic membrane of the PROM group (Fig. 4D). WB analysis revealed that, in comparison with the NORMAL group, the PROM group presented reduced levels of the autophagy-related molecule P62, while the expression levels of LC3B, ATG5, and Beclin-1 were elevated, as shown in Fig. 4E. This finding suggests activated autophagy under PROM conditions.

The extracted fibroblasts were exposed to autophagy-inducing conditions and maintained in a serum-free culture (SS) for a duration of four days. Fluorescence staining revealed that α-SMA expression was increased in the SS group, and the fibroblasts differentiated into highly contracted fusiform myoblasts (Fig. 4H). Autophagy inhibition (CQ) was performed on the cells with chloroquine. The WB results of autophagy related molecules after autophagy inhibition are shown in Fig. 4F. The expression of α-SMA was reduced in the SS group (Fig. 4G), indicating that autophagy can promote fibroblast differentiation.

### 3.5 MALAT1 promotes autophagy

**Fig. 5:**
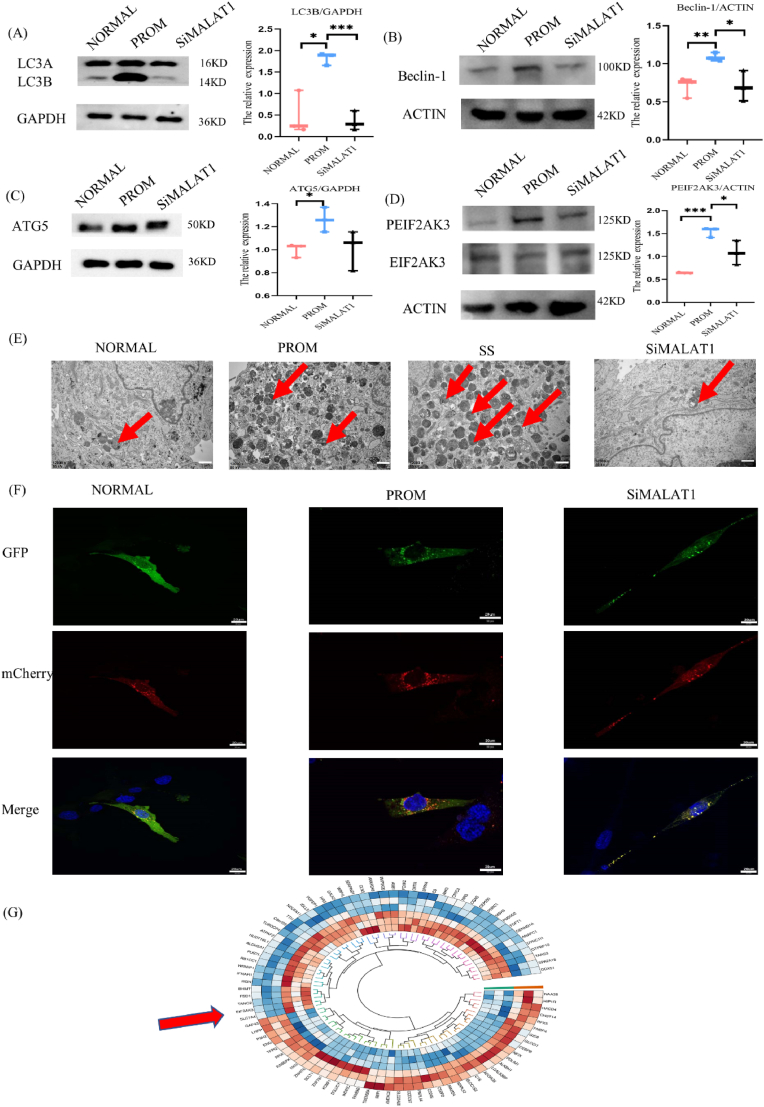
Effect of MALAT1 on autophagy in amniotic fibroblasts (A) WB results showing the expression of LC3 in cells,n = 3. (B) WB results showing the expression of Beclin-1 in cells, n = 3. (C) WB results showing the expression of ATG5 in cells, n = 3. (D) WB results showing the expression of PEIF2AK3 in cells, n = 3. (E) Representative TEM image of a fibroblast with arrows indicating autophagy lysosomes. (F) Detection of autophagy flow in the NORMAL, PROM and SiMALAT1 groups, 60X. (G) Circular clustering heatmap of differentially expressed proteins. Red indicates a relatively high relative expression level, blue indicates a relatively low relative expression level, and the red arrow indicates EIF2AK3.

In this study, we investigated the impact of MALAT1 downregulation on autophagy in amniotic fibroblasts utilizing MALAT1 knockdown. Compared with PROM, MALAT1 knockdown resulted in decreased expression of the autophagy proteins LC3B, Beclin-1, and ATG5 (Fig. 5A, B, C). Through TEM observation of amniotic fibroblasts, we discovered that the PROM group presented an increased prevalence of autophagic lysosomes in the amniotic membrane compared with the NORMAL group. Furthermore, in the SS group, the presence of autophagic lysosomes was also increased, with less autophagy observed in the SiMALAT1 group than in both the PROM and the SS groups. Autophagy was activated in PROM and that knocking down MALAT1 inhibited autophagy (Fig. 5E).

Protein pathway sequencing data analysis revealed that the expression of the autophagy marker EIF2AK3 was reduced in the MALAT1 knockout group (Fig. 5G), which was validated by WB. Compared with that in the PROM group, the expression of PEIF2AK3 in the SiMALAT1 group was decreased (Fig. 5D), which verified the above changes in the protein sequencing pathway. We used mCherry-GFP-LC3 adenovirus vectors to determine the effect of MALAT1 on autophagy flux. When the autolysosome is formed, the green fluorescence is weakened, leaving only the red signal, because the red signal is more stable than the green fluorescence under acidic conditions. Confocal microscopy revealed more red dots in the PROM group than in the NORMAL group, indicating that autophagy was activated. The yellow dots were enriched after MALAT1 knockdown, indicating that autolysosomes formation was hindered. The colocalization of red and green fluorescence in the SiMALAT1 group indicated that autophagic flux was blocked and that MALAT1 promoted autophagy (Fig. 5F).

### 3.6 MALAT1 and CREG1 are combined

**Fig. 6:**
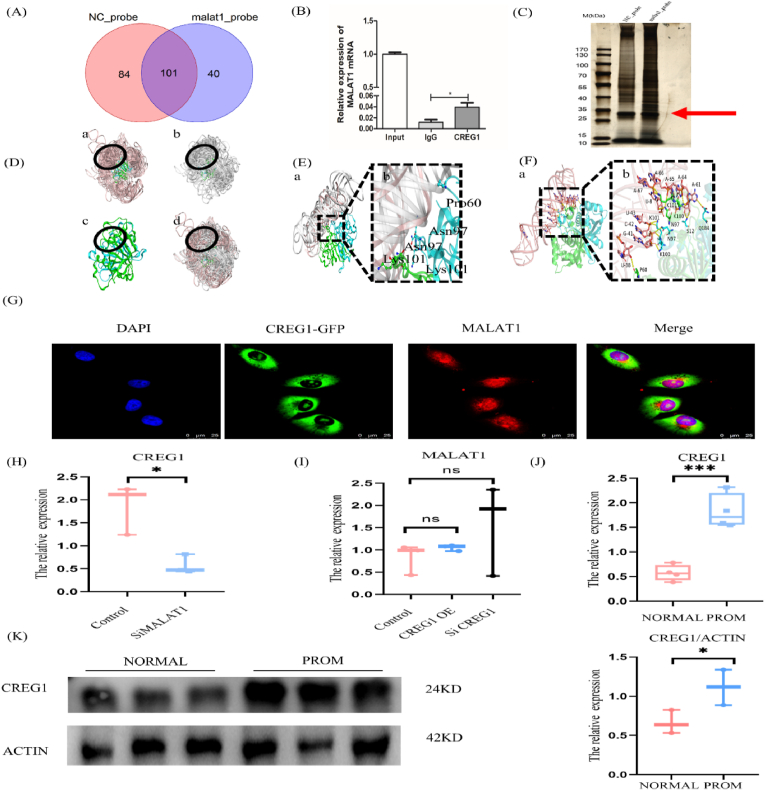
Combination of MALAT1 and CREG1. (A) Venn diagram of enriched proteins in the NC and MALAT1 probe groups. (B) The combination of MALAT1 and CREG1 was confirmed by a RIP assay. (C) RNA PULL DOWN protein silver stain map. (D) Location distribution of the docking results of the MALAT1 conformation and CREG1 dimer (a: MOE docking results; b: HDOCK docking results; c: CREG1 protein dimer; d: MOE, HDOCK docking results and CREG1 protein dimer). (E) The 7 conformations of the conformation concentration region (a: CREG1 protein dimer A and B chains are shown in green and blue, respectively. The conformation of the MOE docking results is light pink, and the HDOCK docking results are gray white. b: Local view of 5 interacting high frequency amino acids and 7 lncRNA conformations of the CREG1 protein dimer). (F) CREG1 and lncRNA interaction modes (a: In the overall view, the A and B chains of the CREG1 protein dimer are shown in green and blue cartoons respectively; LncRNAs are shown in light pink cartoons, amino acids and nucleotides are shown in stick patterns, carbon atoms are in the same color as in cartoon patterns, oxygen atoms are in red; and nitrogen atoms are in blue. Phosphorus atoms are orange. b: local view). (G) Images of MALAT1 and CREG1 fluorescence in situ hybridization. (H) The expression of CREG1 in cells after siMALAT1 was detected by qPCR, n = 3. (I) MALAT1 expression in CREG1-overexpressing and CREG1-knockdown cells was detected by qPCR, n = 3. (J) The expression of CREG1 in human amniotic tissue was detected by qPCR, n = 5. (K) WB results showing the expression of CREG1 in human amniotic tissue, n = 3.

To explore the specific form of MALAT1 function in PROM, we pulled down the proteins that bind to MALAT1 in amniotic tissue by RNA PULLD DOWN, and 40 proteins were detected, indicating that MALAT1 could directly bind to CREG1 (Fig. 6A and C). RIP experiments revealed that CREG1 could indeed bind to MALAT1, demonstrating that MALAT1 specifically enriched CREG1 (Fig. 6B). Molecular docking analysis revealed the presence of multiple binding sites between MALAT1 and CREG1, implying a binding interaction between these two proteins (Fig. 6D, E, and F). Immunofluorescence colocalization of primary amniotic fibroblasts revealed the dual subcellular localization of CREG1 and MALAT1, which is indicative of the interaction of MALAT1 with CREG1 (Fig. 6G). The RNA and protein levels in amniotic membrane tissue also increased in CREG1 in the PROM group (Fig. 6J and K), which is consistent with the trend of MALAT1 in PROM.

The expression of qPCR revealed that CREG1 levels were correspondingly decreased in cells following MALAT1 knockdown (Fig. 6H), and MALAT1 expression was not significantly affected by CREG1 overexpression or knockdown (Fig. 6I), indicating that MALAT1 influences CREG1 expression. These findings highlight the intricate interplay between MALAT1, autophagy, CREG1 and their potential roles in the pathophysiology of PROM.

## 4 Discussion

Research indicates that the causes of PROM include infection, trauma, mechanical injury, poor fetal membrane development, cervical insufficiency, and abnormal intrauterine pressure, among others. However, current research on the etiology and repair mechanisms of PROM is not comprehensive, and no effective targets for repairing PROM have been identified. Therefore, investigating the causes and repair mechanisms of PROM damage is particularly urgent for preventing this condition. Through examinations such as scanning electron microscopy, fluorescence labeling, Sirius staining, and Raman spectroscopy of fetal membrane and amniotic tissues, we indeed detected structural damage in the tissues with PROM, including loose epithelial cell connections and reduced collagen.

To gain a deeper understanding of the biological characteristics and functions of different cell subpopulations within fetal membrane tissues, our research utilized single-cell transcriptome sequencing and various bioinformatics analysis methods. We have preliminarily investigated the cellular clustering, gene expression, physiological functions, and the potential mechanisms and key molecules involved in the regulation of these biological processes by different cell subpopulations within the fetal membrane tissues. The results of single-cell sequencing revealed that the number of fibroblasts in the fetal membrane tissues was the second highest. Fibroblasts are further divided into three subpopulations, among which the number of myofibroblasts significantly increased in the PROM group. The proliferation of fibroblasts and their transformation into activated myofibroblasts is referred to as the activation of fibroblasts. Myofibroblasts are cells that promote wound contraction and the production of collagen fibers, and they are among of the main participants in wound healing, serving as important effectors in normal tissue repair [11]. The literature indicates that myofibroblasts can migrate to the edge of the amniotic wound and release collagen to promote amniotic repair and that the permeability and tension of the fetal membrane are mainly related to the extracellular matrix proteins produced by myofibroblasts [19]. Our research revealed an increase in α-SMA, which represents myofibroblasts, in PROM, confirming the existence of fibroblast activation in PROM. Therefore, studying the fibroblast activation process is crucial for the repair of the fetal membrane.

Our research utilized TEM to identify autophagic vesicles within amniotic tissue. Autophagy is an ancient and highly conserved cellular mechanism that contributes to cellular survival. Autophagy not only provides nutrients to prevent starvation-induced apoptosis but also promotes the activation of fibroblasts and tissue fibrosis [18]. Impaired autophagy is significantly antifibrotic, reducing the transition of fibroblasts to myofibroblasts [20]. Studies have shown that autophagy plays a role in maintaining tissue homeostasis in chorionic cells [21], but there is less research on autophagy in the amnion. The Western blot results suggest that autophagy is indeed activated in the amnion, and this finding was further verified by treating amniotic fibroblasts with an autophagy inhibitor. Compared with the PROM group, the autophagy inhibition group presented a decrease in α-SMA. Therefore, we speculate that there is an autophagic phenomenon in the amnion of tissues with PROM, which can promote the activation of fibroblasts.

By analyzing single-cell sequencing data, we found that the expression of MALAT1 in the amniotic fibroblasts of the PROM group was significantly increased. As a long noncoding RNA of 8076 bases, MALAT1 can form various dynamic conformations within the cell, which can act as a scaffold to facilitate its interaction with different RNA-binding proteins [22] and participate in the recruitment of RNA polymerase II, transcription factors, and cofactors to gene promoters, thereby controlling the transcription of target genes [23]. The literature suggests that MALAT1 also plays an important role in the regulation of autophagy. For example, MALAT1 promotes the proliferation and metastasis of invasive pancreatic cancer by stimulating autophagy and can also activate autophagy and promote cell proliferation in gliomas [24]. MALAT1 in gastric cancer also inhibits autophagy flux and stimulates IL-6 by regulating the PTEN/AKT/mTOR pathway, thereby transforming fibroblasts into myofibroblasts [25]. MALAT1 is abundant in fibroblasts and can promote the migration of human dermal fibroblasts and wound healing [19]. Proteomic sequencing and validation of MALAT1-knockdown cells revealed that the EIF2AK3 pathway was blocked after MALAT1 was knocked down, which is closely related to autophagy, suggesting that MALAT1 can indeed regulate the autophagy pathway. This study revealed via WB that the knockdown of MALAT1 can lead to a decrease in myofibroblasts and an increase in fibroblasts, blocking the autophagy pathway, and suggesting that MALAT1 can affect the autophagy pathway and the activation of fibroblasts. We performed scratch assays, transwell migration assays, and CCK8 proliferation assays on primary fibroblasts. The results indicated that, in comparison with the NORMAL group, the migration capacity of the PROM group was significantly enhanced. Moreover, knockdown of MALAT1 effectively suppressed the migration and proliferation functions of the cells. Interestingly, migration activity appears to be more conducive to membrane repair under PROM conditions.

Building upon this foundation, we conducted RNA PULL DOWN experiments on amniotic tissue to explore how MALAT1 affects autophagy and fibroblast activation. We discovered that MALAT1 primarily interacts with 40 proteins, among which CREG1 plays a significant role in autophagy. CREG1 is a lysosomal protein initially characterized as a transcriptional repressor that antagonizes transcription and cellular transformation induced by the adenovirus E1A oncogene [26]. YAN CH et al. revealed that the overexpression of CREG1 can rapidly lead to the degradation of p62, promoting the occurrence of autophagy [27]. LIU J et al. reported that CREG1, located in the endosome-lysosome compartment, can promote lysosomal biogenesis, acidification, and degradation, thereby accelerating autophagic flux [25]. Therefore, CREG1 is a key mediator of autophagy activation. Our preliminary experimental results indicated that the knockdown/overexpression of CREG1 did not affect the expression of MALAT1, but the knockdown of MALAT1 would lead to the decrease of CREG1. Therefore, we believed that MALAT1 was the upstream of CREG1 and could regulate the expression of CREG1 transcriptional gene and increase the stability of CREG1 [28]. The cell co-localization images showed that MALAT1 and CREG1 mainly co-located in the nucleus, indicating that MALAT1 may induce the expression of downstream autophagy molecules through its interaction with CREG1 in the nucleus. Molecular docking results also confirmed the interaction of MALAT1 and CREG1 dimers. At present, our study is focused on fetal membrane tissue. In order to better diagnose and treat PROM, future studies may focus on detecting MALAT1/CREG1 levels in pregnant women and supplementing with exosomes containing MALAT1 [29] or CREG1 recombinant proteins. This will provide a potential therapeutic target for the prevention and treatment of PROM.

## 5 Conclusion

On the basis of our findings, we conclude that MALAT1 enhances the expression of CREG1 through interaction, thereby regulating autophagy-related molecules, mediating the differentiation of amniotic fibroblasts into myofibroblasts, participating in the repair of the amnion, and subsequently promoting the repair of PROM fetal membrane tissue. This study provides a potential therapeutic target for the prevention and treatment of PROM by elucidating the role of MALAT1 and CREG1 in the regulation of autophagy and fibroblast activation.

## CRediT authorship contribution statement

**Xiaomei Huang:** Writing – review & editing, Writing – original draft, Conceptualization. **Ting Huang:** Writing – review & editing, Methodology, Data curation. **Aixing Chen:** Software, Methodology. **Yong Shao:** Validation, Funding acquisition.

## Ethics approval and consent to participate

The present study was approved by the ethical committee of the First Affiliated Hospital of Chongqing Medical University (Ethical Application Ref: 2022-160). All procedures performed in studies involving human participants were in accordance with the ethical standards of the institutional and national research committees and with the 1964 Helsinki declaration and its later amendments or comparable ethical standards. All participants signed a written informed consent form prior to their participation in this study.

## Availability of data and materials

The datasets supporting the conclusions of this article are included within the article.

## Funding

This work is funded by the National Natural Science Foundation of China (No: 81471473).This work is funded by National Key R&D Program of China (No. 2021YFC2701500, 2021YFC2701501, 2021YFC2701505). These funding supported the design of the study; the collection, analysis, and interpretation of the data; and the writing of the manuscript.

## Declaration of competing interest

The authors declare that they have no competing interests.

## Abbreviations

PROM: Premature rupture of fetal membrane
MALAT1: Metastasis-Associated Lung Adenocarcinoma Transcript 1
CREG1: Human E1A promoter repressor 1
ECM: Extracellular matrix
GO: Gene Ontology
TEM: Transmission Electron Microscopy
CK19: Cytokeratin 19
SS: Serum-free medium
CQ: Chloroquine
RIP: RNA Immunoprecipitation
FISH: Fluorescence in Situ Hybridization
WB: Western Blot
QPCR: Quantitative Real-time polymerase chain reaction
HE: hematoxylin–eosin
DIA: Data Independent Acquisition

## References

[1] Bouvier D., Forest J.C., Blanchon L., Bujold E., Pereira B., Bernard N., Gallot D., Sapin V., Giguère Y. (2019). Risk factors and outcomes of preterm premature rupture of membranes in a cohort of 6968 pregnant women prospectively recruited. J. Clin. Med..

[2] Barinov S.V., Tirskaya Y.I., Kadsyna T.V., Lazareva O.V., Medyannikova I.V., Tshulovski Y.I. (2022). Pregnancy and delivery in women with a high risk of infection in pregnancy. J. Matern. Fetal Neonatal Med..

[3] Weiner E., Barrett J., Zaltz A., Ram M., Aviram A., Kibel M., Lipworth H., Asztalos E., Melamed N. (2019). Amniotic fluid volume at presentation with early preterm prelabor rupture of membranes and association with severe neonatal respiratory morbidity. Ultrasound Obstet. Gynecol..

[4] Pasquier J.C., Doret M. (2008). [Fetal membranes: embryological development, structure and the physiopathology of the preterm premature rupture of membranes]. J. Gynecol. Obstet. Biol. Reprod..

[5] Manabe Y., Himeno N., Fukumoto M. (1991). Tensile strength and collagen content of amniotic membrane do not change after the second trimester or during delivery. Obstet. Gynecol..

[6] Oyen M.L., Calvin S.E., Landers D.V. (2006). Premature rupture of the fetal membranes: is the amnion the major determinant?. Am. J. Obstet. Gynecol..

[7] Arikat S., Novince R.W., Mercer B.M., Kumar D., Fox J.M., Mansour J.M., Moore J.J. (2006). Separation of amnion from choriodecidua is an integral event to the rupture of normal term fetal membranes and constitutes a significant component of the work required. Am. J. Obstet. Gynecol..

[8] Polzin W.J., Brady K. (1998). The etiology of premature rupture of the membranes. Clin. Obstet. Gynecol..

[9] Zhang C., Wang W., Liu C., Lu J., Sun K. (2017). Role of NF-κB/GATA3 in the inhibition of lysyl oxidase by IL-1β in human amnion fibroblasts. Immunol. Cell Biol..

[10] James R., Kesturu G., Balian G. (2008). Chhabra A B Tendon: biology, biomechanics, repair, growth factors, and evolving treatment options. J Hand Surg Am.

[11] Li B., Wang J.H. (2011). Fibroblasts and myofibroblasts in wound healing: force generation and measurement. J. Tissue Viability.

[12] Deb A., Ubil E. (2014). Cardiac fibroblast in development and wound healing. J. Mol. Cell. Cardiol..

[13] Huang X., Liao J., Feng F., Chen S., Liao E., Li D., Dai X., Dong J., Shao Y. (2023). Combined application of exosomes and FPR2 agonist LXA4 in controlling fetal membrane inflammation and promoting fetal membrane tissue repair. Reprod. Sci..

[14] Richardson L.S., Vargas G., Brown T., Ochoa L., Sheller-Miller S., Saade G.R., Taylor R.N., Menon R. (2017). Discovery and characterization of human amniochorionic membrane microfractures. Am. J. Pathol..

[15] Borges L.F., Gutierrez P.S., Marana H.R., Taboga S.R. (2007). Picrosirius-polarization staining method as an efficient histopathological tool for collagenolysis detection in vesical prolapse lesions. Micron.

[16] Auner G.W., Koya S.K., Huang C., Broadbent B., Trexler M., Auner Z., Elias A., Mehne K.C., Brusatori M.A. (2018). Applications of Raman spectroscopy in cancer diagnosis. Cancer Metastasis Rev..

[17] Lu J.W., Lei W.J., Ling L.J., Wang L.Y., Lin Y.K., Zhang F., Li M.D., Pan F., Wang W.S., Sun K. (2022). Cortisol stimulates local progesterone withdrawal through induction of AKR1C1 in human amnion fibroblasts at parturition. Endocrinology.

[18] Bernard M., Yang B., Migneault F., Turgeon J., Dieudé M., Olivier M.A., Cardin G.B., El-Diwany M., Underwood K., Rodier F., Hébert M.J. (2020). Autophagy drives fibroblast senescence through MTORC2 regulation. Autophagy.

[19] Liang Z.H., Pan Y.C., Lin S.S., Qiu Z.Y., Zhang Z. (2020). LncRNA MALAT1 promotes wound healing via regulating miR-141-3p/ZNF217 axis. Regen. Ther..

[20] Zhang Y., Shen L., Zhu H., Dreissigacker K., Distler D., Zhou X., Györfi A.H., Bergmann C., Meng X., Dees C., Trinh-Minh T., Ludolph I., Horch R., Ramming A., Schett G., Distler J.H.W. (2020). PGC-1α regulates autophagy to promote fibroblast activation and tissue fibrosis. Ann. Rheum. Dis..

[21] Avagliano L., Massa V., Zullino S., Doi P., Marconi A.M., Ferrazzi E., Bulfamante G. (2017). P Inflammation modulates LC3 expression in human preterm delivery. J. Matern. Fetal Neonatal Med..

[22] Ghosh A., Pandey S.P., Joshi D.C., Rana P., Ansari A.H., Sundar J.S., Singh P., Khan Y., Ekka M.K., Chakraborty D, Maiti S. (2023). Identification of G-quadruplex structures in MALAT1 lncRNA that interact with nucleolin and nucleophosmin. Nucleic Acids Res..

[23] Zhao N., Hua W., Liu Q., Wang Y., Liu Z., Jin S., Wang B., Pang Y., Qi J., Song Y. (2023). MALAT1 knockdown alleviates the pyroptosis of microglias in diabetic cerebral ischemia via regulating STAT1 mediated NLRP3 transcription. Mol. Med..

[24] Wang S., Han X., Mao Z., Xin Y., Maharjan S., Zhang B. (2019). MALAT1 lncRNA induces autophagy and protects brain microvascular endothelial cells against oxygen-glucose deprivation by binding to miR-200c-3p and upregulating SIRT1 expression. Neuroscience.

[25] Wang Z., Wang X., Zhang T., Su L., Liu B., Zhu Z., Li C. (2021). LncRNA MALAT1 promotes gastric cancer progression via inhibiting autophagic flux and inducing fibroblast activation. Cell Death Dis..

[26] Veal E., Eisenstein M., Tseng Z.H., Gill G. (1998). A cellular repressor of E1A-stimulated genes that inhibits activation by E2F. Mol. Cell Biol..

[27] Yan C.H., Li Y., Tian X.X., Zhu N., Song H.X., Zhang J., Sun M.Y., Han Y.L. (2015). CREG1 ameliorates myocardial fibrosis associated with autophagy activation and Rab7 expression. Biochim. Biophys. Acta.

[28] Miyagawa R., Tano K., Mizuno R., Nakamura Y., Ijiri K., Rakwal R., Shibato J., Masuo Y., Mayeda A., Hirose T., Akimitsu N. (2012). Identification of cis- and trans-acting factors involved in the localization of MALAT-1 noncoding RNA to nuclear speckles. RNA.

[29] Cooper D.R., Wang C., Patel R., Trujillo A., Patel N.A., Prather J., Gould L.J., Wu M.H. (2018). Human adipose-derived stem cell conditioned media and exosomes containing MALAT1 promote human dermal fibroblast migration and ischemic wound healing. Adv. Wound Care.

